# Evaluation of the effect of different sitting assistive devices in reclining wheelchair on interface pressure

**DOI:** 10.1186/s12938-017-0398-8

**Published:** 2017-08-29

**Authors:** Chun-Ting Li, Kuo-Yuan Huang, Chien-Feng Kung, Yen-Nien Chen, Yen-Ting Tseng, Kuen-Horng Tsai

**Affiliations:** 1 Applied Biomechanics Laboratory, Graduate Institute of Mechatronic System Engineering, National University of Tainan, No. 33, Sec. 2, Shu-Lin St., West Central Dist., Tainan, 70005 Taiwan; 2 Department of Orthopedics, College of Medicine, National Cheng Kung University Hospital, No.138, Sheng-Li Rd., North Dist., Tainan, 70403 Taiwan; 3 Graduate Institute & Department of Information Management, National Kaohsiung University of Applied Sciences, No. 415, Chien-Kung Rd., Sanmin Dist., Kaohsiung, 80778 Taiwan; 4 Department of BioMedical Engineering, National Cheng Kung University, No. 1, University Rd., East Dist., Tainan, 70101 Taiwan

**Keywords:** Wheelchair, Pressure ulcer, Sitting assistive device, Interface pressure

## Abstract

**Background:**

Reclining wheelchair users often add one or more sitting assistive devices to their wheelchairs, but the effect of these additional sitting assistive devices on the risk of pressure ulcers has rarely been investigated. This study examined the four modes of reclining wheelchair without and with different sitting assistive devices, namely the back reclined mode, the lumbar support with back reclined mode, the femur upward with back reclined mode, and the lumbar support with femur upward with back reclined mode, in terms of their effects on human-wheelchair interface pressure.

**Methods:**

This study recruited 16 healthy participants to undergo the aforementioned four modes in random order and have their human-wheelchair interface pressure measured. The initial setting of experimental reclining wheelchair backrest was pushed backward to reach a 150° recline. The data on interface pressure were collected for 5 s while the participant maintained a stable sitting position. The contact area, average pressure, and peak pressure on the back area, ischial area, and femur area were recorded and calculated.

**Results:**

Among all tested modes, the lumbar support with femur upward with back reclined mode provided the most significant reduction in stress load on the ischial area (*P* ≤ 0.010) and shifted part of the load to the femur area (*P* ≤ 0.009).

**Conclusions:**

This study quantified the effects of and differences between various reclining wheelchair–sitting assistive device combination modes. These findings are useful for the decision-making processes of rehabilitation physicians, wheelchair users, and manufacturers.

## Background

Pressure ulcers are an essential clinical topic. Previous literature reviews have indicated that pressure ulcer prevalence in intensive care settings ranges from 4 to 49% and incidence ranges from 3.8 to 12.4% [1]. It influences patients’ recovery time, life quality, treatment cost, or even develop a life-threatening infection [2–4]. Pressure ulcers are most commonly induced by long-term stress exerted on the bony prominence, which causes the blood vessels surrounding the peripheral soft tissues to compress, thereby influencing blood flow and nutrient supply and causing cell hypoxia and necrosis [3–5]. Previous studies have reported that a prolonged static sitting load increases the risk of pressure ulcers [3, 4]. In particular, patients who experience lower extremity disabilities caused by brain or spinal disorders and require the prolonged use of a wheelchair are at a high risk of pressure ulcers [4].

Reclining wheelchairs are often used by people with brain or spinal disorders. The reclining function of the wheelchair redistributes the human-wheelchair interface pressure by partially shifting it from the buttocks area to the back area, which not only decreases the problem of stress concentration at the ischial tuberosities (ITs) but also increases the muscle and skin perfusion of weight-bearing soft tissues [6–8]. The pressure ulcer risk-reduction function of reclining wheelchairs has been demonstrated by previous studies [9].

Researchers have developed various sitting assistive devices (SADs) such as lumbar supports and cushions [4, 10–18]. These devices can be used independently on different types of wheelchairs. Some studies have shown that lumbar supports can reduce stress on the intervertebral discs by creating lumbar lordosis at the waist and shifting part of the stress from the buttocks area to the lumbar support that the back area is leaning on [13–16, 19]. Furthermore, cushions correct the problem of stress concentration at the ITs by redistributing stress on the buttocks and thighs [4, 12, 18]. Nonetheless, the effects of most of these SADs have only been validated using the standard wheelchair design.

A noteworthy phenomenon has been detected in clinical observation: numerous reclining wheelchair users have selected one or more than one of these SADs and positioned them on their wheelchairs. In addition, some reclining wheelchair manufacturers even equip their wheelchair products with some of these SADs to increase the market competitiveness of their products. However, few studies have examined whether incorporating these SADs into reclining wheelchairs exerts any effect on human-wheelchair interface pressure, which are critical mechanisms of pressure ulcers.

**Fig. 1:**
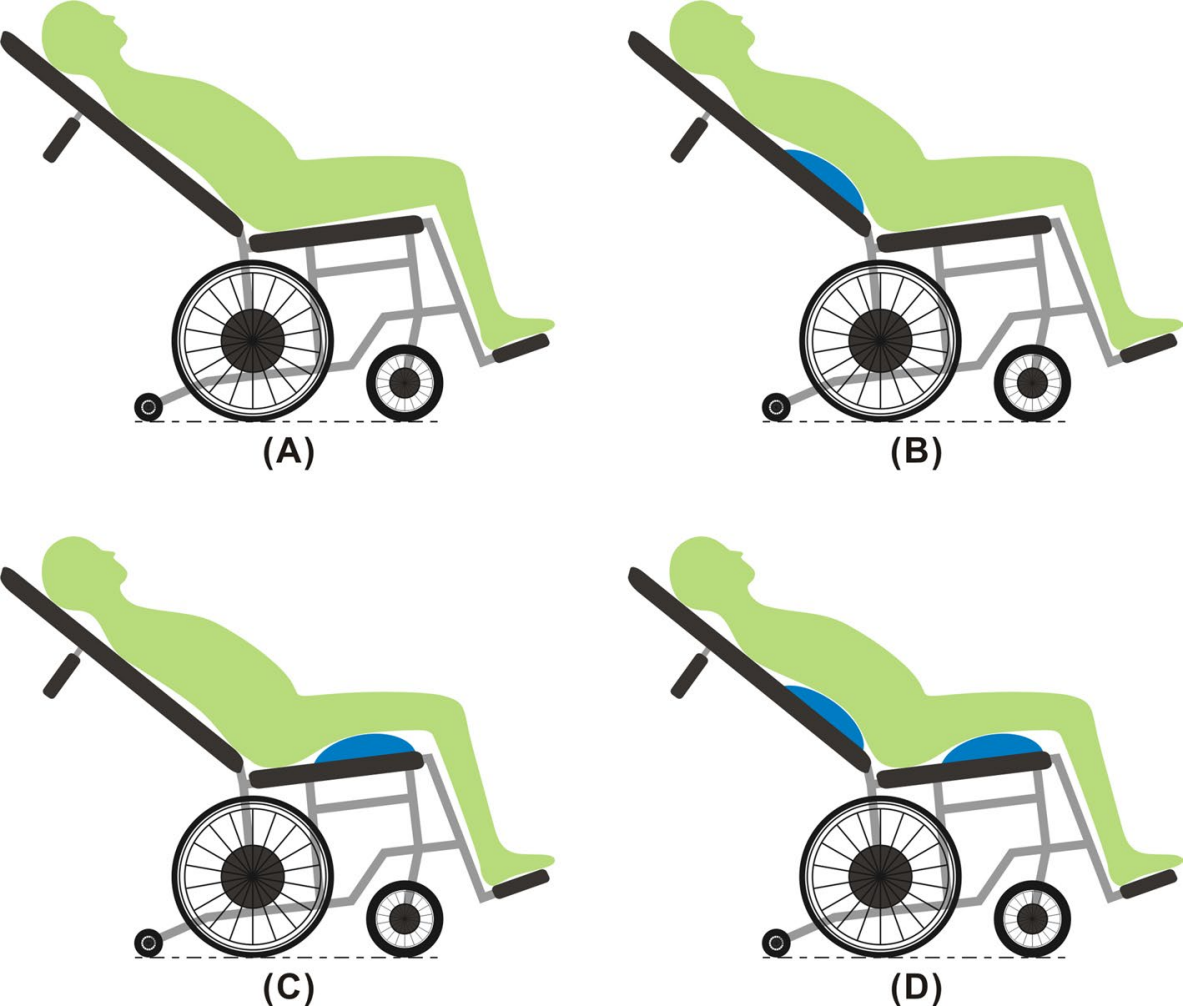
Four different tested modes. Including: **a** back reclined mode (BRM), **b** lumbar support with back reclined mode (LBM), **c** femur upward with back reclined mode (FBM), and **d** lumbar support with femur upward with back reclined mode (LFBM)

Therefore, the present study examined the four modes of reclining wheelchair without and with different SADs (Fig. 1), namely the back reclined mode (BRM), lumbar support with back reclined mode (LBM), femur upward with back reclined mode (FBM), and lumbar support with femur upward with back reclined mode (LFBM), in terms of their effects on human-wheelchair interface pressure.

## Methods

### Subjects

Healthy participants were recruited for this study. During the recruitment period, we screened out those with identifiable spinal pathologies, musculoskeletal disorders, and movement disorders. All participants read and signed an informed consent form, which explained the objectives and the experimental protocol. This study was approved by the Institutional Review Board of National Cheng Kung University Hospital. All experiments were performed in accordance with relevant guidelines and regulations.

### Modes

**Fig. 2:**
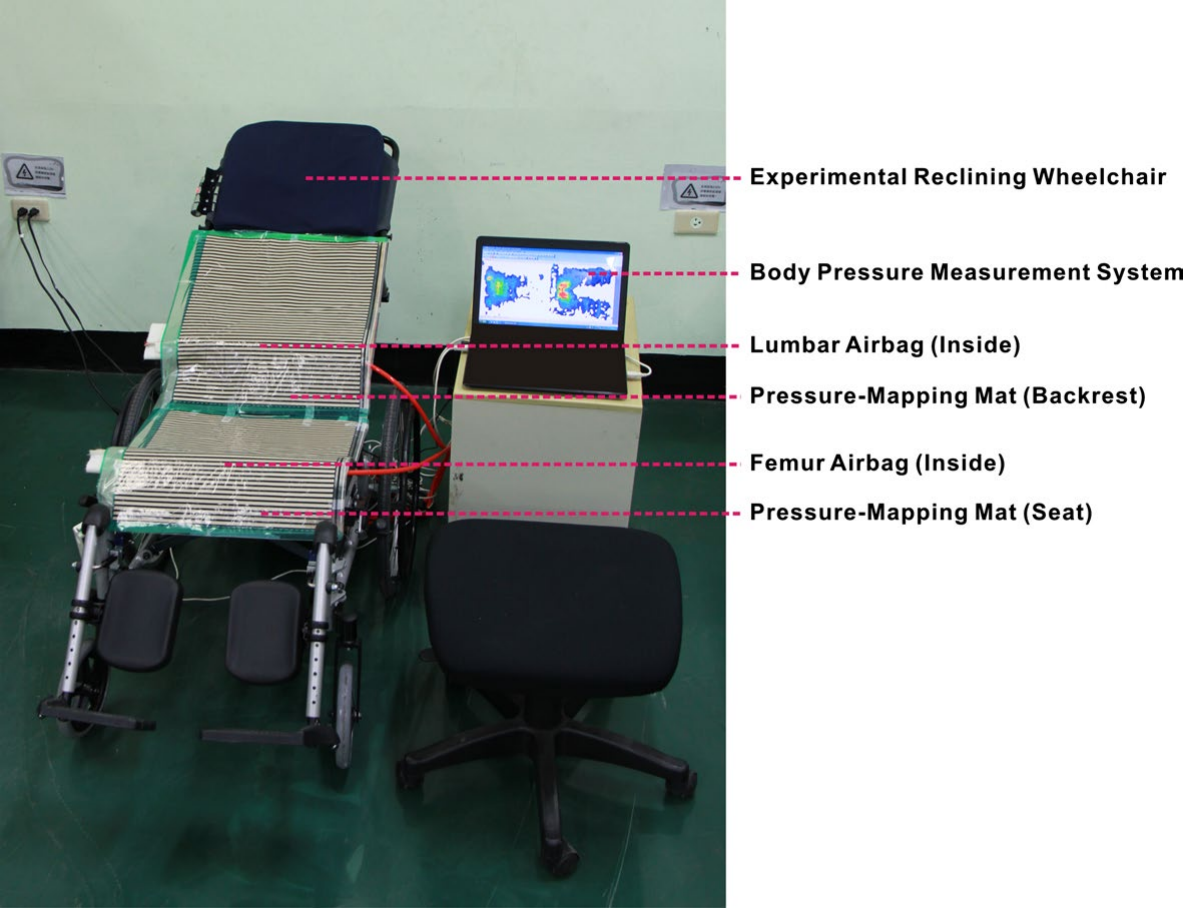
Experimental setup. The picture shows the experimental setup with experimental reclining wheelchair, lumbar airbag (inside), femur airbag (inside), pressure-mapping mat (backrest), pressure-mapping mat (seat), and body pressure measurement system

An experimental reclining wheelchair was used in this study. Its backrest had a reclining range between 90° and 160°. This wheelchair could be equipped with two airbags, one on the backrest for the lumbar area and the other on the seat for the femur area (Fig. 2). The airbag measured 40 × 23 cm^2^, with an adjustable thickness of 0–4 cm. Foam with a thickness of 1 cm was attached to each backrest and seat to reduce discomfort resulting from contact between the body and the uneven surfaces of the backrest and the seat. The researchers paired the wheelchair without and with the above-mentioned airbags to create four modes (Fig. 1): (1) BRM: the wheelchair backrest was pushed backward to reach a 150° recline [20, 21]. (2) LBM: the backrest of the wheelchair was pushed backward to reach a 150° recline, while a lumbar airbag, fully inflated to a thickness of 4 cm, was placed at the L3 spinal segment of the participant [20–23]. (3) FBM: the backrest of the wheelchair was pushed backward to reach a 150° recline, and a femur airbag, fully inflated to a thickness of 4 cm, was placed at the midpoint of the thighs of the participant [19–21]. (4) LFBM: the backrest of the wheelchair was pushed backward reach a 150° recline and both lumbar and femur airbags were used, the lumbar airbag, fully inflated to a thickness of 4 cm, was placed at the L3 segment of the participant, whereas the femur airbag, also fully inflated to a thickness of 4 cm, was placed at the midpoint of the thighs of the participant [19–23].

### Procedures

First, the backrest of the experimental reclining wheelchair was adjusted to form a 150° angle with the seat [20]. Then, the seat of wheelchair remained still while the seat-footrest angle was adjusted to 120° angle [20]. Next, the study participants took turns sitting in the experimental reclining wheelchair. When sitting, participants were asked to lean their upper bodies against the backrest, relax their arms at their sides, keep their thighs as parallel with the ground as possible, and space their feet at approximately shoulder width on the footplates. The length of the footrests should be adjusted according to the calves and feet lengths of the participant to ensure that the sole is in full contact with the footplates. Participants were then asked to test the four above-mentioned modes while interface pressure was measured. The sequence of the four tested modes was randomly drawn by each participant. The data on interface pressure were collected for 5 s while the participant maintained a stable sitting position. The participants were then requested to stand for 1 min between tested modes.

### Measurement

Two pressure-mapping mats (Body Pressure Measurement System; Tekscan Inc, South Boston, Massachusetts, USA) were fixed with ties to the experimental reclining wheelchair: one to the backrest and the other to the seat (Fig. 2). They were used to measure human-wheelchair interface pressure. Both pressure-mapping mats measured 487.68 mm × 426.72 mm × 0.33 mm, and they were thin and flexible. The mats comprised 2016 (48 × 42) measuring cells, each with dimensions of 10.16 × 10.16 mm^2^. The interface pressure parameters were calculated using the body pressure measurement system research software (BPMS, version 7.02C; Tekscan Inc, South Boston, Massachusetts, USA). Data were sampled at a frequency of 30 Hz. The contact area (CA), average pressure (AP), and peak pressure (PP) on the back area (BCA, BAP, and BPP), the ischial area (ICA, IAP, and IPP), and the femur area (FCA, FAP, and FPP) were recorded and calculated. Numerous studies have used these parameters to determine the risk of pressure ulcers [13, 20, 24]. The CA refers to the size of the interface pressure contact area between the human body and wheelchair. The AP refers to the stress level of the interface pressure contact area between the human body and wheelchair. The PP refers to the stress concentration location and force load of the interface pressure contact area between the human body and wheelchair. These parameters were positively correlated with the risk of pressure ulcers.

### Analyses

The Statistical Package for the Social Sciences (SPSS, version 17; SPSS Institute, Chicago, IL, USA) was used for all statistical analysis. All parameters (BCA, BAP, BPP, ICA, IAP, IPP, FCA, FAP, and FPP) were compared in the different tested modes (BRM, LBM, FBM, and LFBM) by using a Friedman test. A post hoc test (Wilcoxon signed-rank test) was used for detecting statistically significant differences in the dependent variables across the tests. Statistical significance was set at *P* < 0.05.

## Results

A total of 16 healthy participants (7 men, 9 women; age, 22.6 ± 1.5 years old; weight, 61.9 ± 12.3 kg; height, 166.4 ± 9.0 cm; body mass index, 22.2 ± 3.0 kg/m^2^) were recruited for this study. All the participants completed the pressure measurements using the experimental reclining wheelchair in BRM, LBM, FBM, and LFBM. No participants reported adverse reactions to the experimental procedures.

The pressure measurements on the back area are shown in Table 1. When compared with BRM, LBM and FBM appeared no significant differences in BCA, BAP, and BPP values were observed. When compared with BRM, LFBM appeared to yield significantly lower BPP (*P* = 0.023) values, but no significant differences in BCA and BAP values were observed. When compared with LBM, FBM and LFBM appeared no significant differences in BCA, BAP, and BPP values were observed. When compared with FBM, LFBM appeared no significant differences in BCA, BAP, and BPP values were observed.

**Table 1:** Results of pressure measurement on the back area

Modes	BCA (cm^2^)	BAP (kPa)	BPP (kPa)
BRM	152.44 ± 88.02	2.59 ± 0.31	4.71 ± 1.35
P (BRM vs. LBM)	0.518	0.063	0.162
P (BRM vs. FBM)	0.796	0.550	0.305
P (BRM vs. LFBM)	0.918	0.132	*0.023*
LBM	166.19 ± 93.59	2.39 ± 0.20	4.10 ± 0.80
P (LBM vs. FBM)	0.642	0.213	0.576
P (LBM vs. LFBM)	0.938	0.569	0.407
FBM	153.69 ± 70.73	2.53 ± 0.35	4.44 ± 1.50
P (FBM vs. LFBM)	0.959	0.300	0.255
LFBM	163.06 ± 83.18	2.42 ± 0.30	3.87 ± 1.26
P (BRM vs. LBM vs. FBM vs. LFBM)	0.913	0.178	*0.019*

Comparison of mean contact area (CA), average pressure (AP), and peak pressure (PP) on the back area (BCA, BAP, and BPP) across four different modes, which include back reclined mode (BRM), lumbar support with back reclined mode (LBM), femur upward with back reclined mode (FBM), and lumbar support with femur upward with back reclined mode (LFBM). Values are mean ± standard deviation (N = 16)

*P* is given for statistical significance

The pressure measurements on the ischial area are shown in Table 2. When compared with BRM, LBM appeared to yield significantly lower IPP (*P* = 0.001) values, but no significant differences in ICA and IAP values were observed. When compared with BRM, FBM appeared to yield significantly lower ICA (*P* < 0.001) and IPP (*P* = 0.021) values, but no significant differences in IAP values were observed. When compared with BRM, LFBM appeared to yield significantly lower ICA (*P* = 0.001), IAP (*P* = 0.003), and IPP (*P* < 0.001) values. When compared with LBM, FBM appeared to yield significantly lower ICA (*P* = 0.001) values, but no significant differences in IAP and IPP values were observed. When compared with LBM, LFBM appeared to yield significantly lower ICA (*P* = 0.001), IAP (*P* = 0.010), and IPP (*P* = 0.023) values. When compared with FBM, LFBM appeared to yield significantly lower IAP (*P* = 0.004) and IPP (*P* = 0.001) values, but no significant differences in ICA values were observed.

**Table 2:** Results of pressure measurement on the ischial area

Modes	ICA (cm^2^)	IAP (kPa)	IPP (kPa)
BRM	438.06 ± 119.12	4.05 ± 1.00	21.67 ± 11.93
P (BRM VS. LBM)	0.301	0.074	*0.001*
P (BRM VS. FBM)	*<0.001*	0.172	*0.021*
P (BRM VS. LFBM)	*0.001*	*0.003*	*<0.001*
LBM	417.25 ± 122.13	3.74 ± 0.95	15.19 ± 6.82
P (LBM VS. FBM)	*0.001*	0.796	0.063
P (LBM VS. LFBM)	*0.001*	*0.010*	*0.023*
FBM	329.06 ± 118.24	3.81 ± 1.01	18.97 ± 10.24
P (FBM VS. LFBM)	0.393	*0.004*	*0.001*
LFBM	340.19 ± 134.36	3.33 ± 0.89	13.18 ± 8.33
P (BRM VS. LBM VS. FBM VS. LFBM)	*<0.001*	*0.002*	*<0.001*

Comparison of mean contact area (CA), average pressure (AP), and peak pressure (PP) on the ischial area (ICA, IAP, and IPP) across four different modes, which include back reclined mode (BRM), lumbar support with back reclined mode (LBM), femur upward with back reclined mode (FBM), and lumbar support with femur upward with back reclined mode (LFBM). Values are mean ± standard deviation (N = 16)

*P* is given for statistical significance

The pressure measurements on the femur area are shown in Table 3. When compared with BRM, LBM appeared no significant differences in FCA, FAP, and FPP values were observed. When compared with BRM, FBM and LFBM appeared to yield significantly higher FCA (*P* < 0.001), FAP (*P* < 0.001), and FPP (*P* < 0.001) values. When compared with LBM, FBM and LFBM appeared to yield significantly higher FCA (*P* = 0.001), FAP (*P* < 0.001), and FPP (*P* < 0.001) values. When compared with FBM, LFBM appeared to yield significantly higher FAP (*P* = 0.009) values, but no significant differences in FCA and FPP values were observed.

**Table 3:** Results of pressure measurement on the femur area

Modes	FCA (cm^2^)	FAP (kPa)	FPP (kPa)
BRM	178.69 ± 92.10	1.77 ± 0.51	2.80 ± 0.73
P (BRM vs. LBM)	0.776	0.804	0.117
P (BRM vs. FBM)	*<0.001*	*<0.001*	*<0.001*
P (BRM vs. LFBM)	*<0.001*	*<0.001*	*<0.001*
LBM	171.38 ± 102.80	1.77 ± 0.48	2.65 ± 0.69
P (LBM vs. FBM)	*0.001*	*<0.001*	*<0.001*
P (LBM vs. LFBM)	*0.001*	*<0.001*	*<0.001*
FBM	285.06 ± 61.64	3.62 ± 0.77	8.91 ± 1.98
P (FBM vs. LFBM)	0.278	*0.009*	0.105
LFBM	299.44 ± 65.58	4.02 ± 0.74	10.13 ± 2.25
P (BRM vs. LBM vs. FBM vs. LFBM)	*<0.001*	*<0.001*	*<0.001*

Comparison of mean contact area (CA), average pressure (AP), and peak pressure (PP) on the femur area (FCA, FAP, and FPP) across four different modes, which include back reclined mode (BRM), lumbar support with back reclined mode (LBM), femur upward with back reclined mode (FBM), and lumbar support with femur upward with back reclined mode (LFBM). Values are mean ± standard deviation (N = 16)

*P* is given for statistical significance

## Discussion

Many factors can increase the risk of pressure ulcers [3–5, 25–27]. Wheelchair seating systems are a critical concern in this regard because of their impact on human-wheelchair interface pressure [4, 13, 24, 28]. When sitting in a standard wheelchair, the user’s buttocks area will bear most of their body weight, and the concentration of stress at the ITs and surrounding soft tissues may aggravate the risk of pressure ulcers [3, 4]. Experiments have shown that reclining wheelchairs are helpful for reducing stress concentration in the IT region [6–8]. Furthermore, to reduce the sitting load, past studies have proposed numerous SADs, such as lumbar supports or cushions, and demonstrated their positive effect on interface pressure [4, 10–18]. Nonetheless, few studies have paired these SADs with reclining wheelchairs to test if any of the combinations can change human-wheelchair interface pressure. Therefore, the present study examined the four modes of reclining wheelchair without and with different SADs, BRM, LBM, FBM, and LFBM; quantified their effects on human-wheelchair interface pressure; and analyzed these effects and the differences between the four tested modes.

No significant difference was found in BCA among the all tested modes. This finding indicates that the four tested modes did not significantly alter the risk of pressure ulcers on the back contact area. No significant differences were found in BAP or BPP among the all tested modes, except for the BPP of the LFBM was significantly smaller than that of BRM. This result might be caused by the intervention of the femur upward devices, which tighten the hamstrings, cause posterior pelvis rotation, increase the reaction force between the waist and lumbar support devices, transfer the stress at the scapula or sacral area (i.e., the scapula or sacral area, where stress is easily concentrated) to the waist area, thereby reducing the BPP of the LFBM. According to the aforementioned description, there was no evidence to indicate that the tested modes could significantly affect the stress load on the back area.

Regarding the pressure on the ICA, that of the FBM and LFBM were significantly smaller than those of the other tested modes, but no significant difference was found between these two modes. This finding demonstrates that the FBM and LFBM can significantly reduce the risk of pressure ulcers on the ischial contact area. Compared with the other tested modes, the LFBM had a significantly smaller IAP, suggesting that LFBM can significantly reduce the stress load on the ischial area. No significant difference in IAP was found among the BRM, LBM, and FBM. As a result, the use of none or only one of the SADs (lumbar support or femur upward devices) did not significantly alter the stress load on the ischial area. Compared with other tested modes, the LFBM had a significantly smaller IPP, whereas the BRM had a significantly larger IPP. This result implies that the LFBM can significantly reduce the stress concentration at the ischial area. For LBM and FBM, no significant IPP difference was found, suggesting that using only one of the SADs (lumbar support or femur upward devices) cannot significantly affect the stress concentration in the ischial area.

The FBM and LFBM demonstrated a significantly greater FCA than other tested modes did, and no significant difference was found between the FBM and LFBM. This finding indicates that both the FBM and LFBM can significantly increase the femur contact area at risk of pressure ulcers. Among all tested modes, the LFBM had significantly greater FAP; the FBM had the second-highest measurement. No significant difference in FAP was found in the other two tested modes. This demonstrates that the LFBM can significantly increase the stress load on the femur area. Among all tested modes, the FBM and LFBM demonstrated a significantly greater FPP, and no significant difference was found between the FBM and LFBM. Furthermore, no significant difference in FPP was found between the other two tested modes. These results imply that FBM and LFBM can significantly concentrate stress at the femur area.

According to these results, the LFBM was the most effective tested mode for reducing the stress load on the ischial area, and it shifted part of the stress to the femur area. This result was possibly due to the simultaneous use of the lumbar support devices and femur upward devices by the LFBM. The intervention of the lumbar support devices caused the spine to form lordosis and increase its reaction force. The intervention of the femur upward devices compressed the soft tissue below the femur and increased the tissue reaction force, which enabled the buttocks to be lifted and a portion of the stress to be transferred from the ischial area to the femur area. Previous studies have shown that the femur area has a higher pressure tolerance than the ischial area does [24, 29]. Therefore, the risk of pressure ulcers can be reduced by shifting pressure from the ischial area, which is more susceptible to pressure ulcers, to the femur area, which has a higher stress tolerance. In clinical applications, the somatotypes of different wheelchair users should be considered to optimize the sizes of the lumbar support devices and femur upward devices.

This research had several limitations. Nondisabled participants were recruited instead of real wheelchair users, and the present study was a short-term evaluation rather than a long-term follow-up investigation. Therefore, if applying the study results to wheelchair users, their different pathological characteristics should be considered to ensure feasibility. In the future, researchers can use this study as a reference for a long-term follow-up study involving wheelchair users. In addition, because of the limitations of the measurement instruments, the pressure mapping mats could be used only to measure the normal stress and not the shear force or partial forces in other axial components. Therefore, this study had the following limitations of measurement: (1) Because the backrest and seat had different orientations, the larger shear force potentially experienced by the back could not be measured. (2) In a sitting position, a person’s hamstrings pull the pelvis and lead to posterior pelvis rotation, which causes the ischial tuberosities to generate a shear force that could not be measured. Accordingly, we were unable to provide clear conclusions regarding pressure variations in different areas within one mode.

## Conclusion

The present study found that none of the modes tested here significantly affected the stress load on the back area. Neither the lumbar support device alone nor the femur upward device alone significantly altered the pressure on the ischial area. The LFBM was the most effective in reducing the stress load on the ischial area and shifting part of the stress to the femur area. The results of this study will be a helpful reference for rehabilitation physicians, wheelchair users, and manufacturers.

## Abbreviations

ITs: ischial tuberosities
SADs: sitting assistive devices
BRM: the back reclined mode
LBM: lumbar support with back reclined mode
FBM: femur upward with back reclined mode
LFBM: lumbar support with femur upward with back reclined mode
BCA: contact area on the back area
BAP: average pressure on the back area
BPP: peak pressure on the back area
ICA: contact area on the ischial area
IAP: average pressure on the ischial area
IPP: peak pressure on the ischial area
FCA: contact area on the femur area
FAP: average pressure on the femur area
FPP: peak pressure on the femur area

## References

[1] Shahin ES, Dassen T, Halfens RJ (2008). Pressure ulcer prevalence and incidence in intensive care patients: a literature review. Nurs Crit Care.

[2] Bhattacharya S, Mishra RK (2015). Pressure ulcers: current understanding and newer modalities of treatment. Indian J Plast Surg.

[3] Wilkins LW (2007). Skillmasters: wound care.

[4] Zacharkow D (1988). Posture: sitting, standing, chair design, and exercise.

[5] Stekelenburg A, Gawlitta D, Bader DL, Oomens CW (2008). Deep tissue injury: how deep is our understanding?. Arch Phys Med Rehabil.

[6] Jan YK, Crane BA, Liao F, Woods JA, Ennis WJ (2013). Comparison of muscle and skin perfusion over the ischial tuberosities in response to wheelchair tilt-in-space and recline angles in people with spinal cord injury. Arch Phys Med Rehabil.

[7] Jan YK, Jones MA, Rabadi MH, Foreman RD, Thiessen A (2010). Effect of wheelchair tilt-in-space and recline angles on skin perfusion over the ischial tuberosity in people with spinal cord injury. Arch Phys Med Rehabil.

[8] Jan YK, Liao F, Jones MA, Rice LA, Tisdell T (2013). Effect of durations of wheelchair tilt-in-space and recline on skin perfusion over the ischial tuberosity in people with spinal cord injury. Arch Phys Med Rehabil.

[9] Groah SL, Schladen M, Pineda CG, Hsieh CH (2015). Prevention of pressure ulcers among people with spinal cord injury: a systematic review. PM R.

[10] Adams MA (2012). The biomechanics of back pain.

[11] Andersson GB, Murphy RW, Ortengren R, Nachemson AL (1979). The influence of backrest inclination and lumbar support on lumbar lordosis. Spine (Phila Pa 1976).

[12] Conine TA, Hershler C, Daechsel D, Peel C, Pearson A (1994). Pressure ulcer prophylaxis in elderly patients using polyurethane foam or Jay wheelchair cushions. Int J Rehabil Res.

[13] Li CT, Chen CH, Chen YN, Chang CH, Tsai KH (2015). Biomechanical evaluation of a novel wheelchair backrest for elderly people. Biomed Eng Online.

[14] Li CT, Chen YN, Chang CH, Tsai KH (2014). The effects of backward adjustable thoracic support in wheelchair on spinal curvature and back muscle activation for elderly people. PLoS ONE.

[15] Makhsous M, Lin F, Bankard J, Hendrix RW, Hepler M, Press J (2009). Biomechanical effects of sitting with adjustable ischial and lumbar support on occupational low back pain: evaluation of sitting load and back muscle activity. BMC Musculoskelet Disord.

[16] Makhsous M, Lin F, Hendrix RW, Hepler M, Zhang LQ (2003). Sitting with adjustable ischial and back supports: biomechanical changes. Spine (Phila Pa 1976).

[17] McGill SM, Fenwick CM (2009). Using a pneumatic support to correct sitting posture for prolonged periods: a study using airline seats. Ergonomics.

[18] Stockton L, Rithalia S (2008). Is dynamic seating a modality worth considering in the prevention of pressure ulcers?. J Tissue Viability.

[19] Li CT, Peng YT, Tseng YT, Chen YN, Tsai KH (2016). Comparing the effects of different dynamic sitting strategies in wheelchair seating on lumbar-pelvic angle. BMC Musculoskelet Disord.

[20] Huang HC, Yeh CH, Chen CM, Lin YS, Chung KC (2011). Sliding and pressure evaluation on conventional and V-shaped seats of reclining wheelchairs for stroke patients with flaccid hemiplegia: a crossover trial. J Neuroeng Rehabil.

[21] Sprigle S, Maurer C, Soneblum SE (2010). Load redistribution in variable position wheelchairs in people with spinal cord injury. J Spinal Cord Med.

[22] De Carvalho DE, Callaghan JP (2012). Influence of automobile seat lumbar support prominence on spine and pelvic postures: a radiological investigation. Appl Ergon.

[23] Reed MP, Schneider LW. Lumbar support in auto seats: conclusions from a study of preferred driving posture. SAE Technical Paper Series 960478; 1996. p. 19–28.

[24] Makhsous M, Rowles DM, Rymer WZ, Bankard J, Nam EK, Chen D, Lin F (2007). Periodically relieving ischial sitting load to decrease the risk of pressure ulcers. Arch Phys Med Rehabil.

[25] Latifa K, Sondess S, Hajer G, Manel BHM, Souhir K, Nadia B, Abir J, Salima F, Abdelhedi M (2016). Evaluation of physiological risk factors, oxidant-antioxidant imbalance, proteolytic and genetic variations of matrix metalloproteinase-9 in patients with pressure ulcer. Sci Rep.

[26] Maklebust J (1987). Pressure ulcers: etiology and prevention. Nurs Clin North Am.

[27] Visscher M, Taylor T (2014). Pressure ulcers in the hospitalized neonate: rates and risk factors. Sci Rep.

[28] van Geffen P, Reenalda J, Veltink PH, Koopman BF (2009). Decoupled pelvis rotation in sitting: a passive motion technique that regulates buttock load associated with pressure ulcer development. J Biomech.

[29] Bennett L, Kavner D, Lee BY, Trainor FS, Lewis JM (1981). Skin blood flow in seated geriatric patients. Arch Phys Med Rehabil.

